# Exploring the Meaning of Organizational Purpose at a New Dawn: The Development of a Conceptual Model Through Expert Interviews

**DOI:** 10.3389/fpsyg.2021.675543

**Published:** 2021-05-17

**Authors:** Ramon van Ingen, Pascale Peters, Melanie De Ruiter, Henry Robben

**Affiliations:** ^1^Center for Strategy, Organization and Leadership, Nyenrode Business University, Breukelen, Netherlands; ^2^Center for Marketing & Supply Chain Management, Nyenrode Business University, Breukelen, Netherlands

**Keywords:** organizational purpose, theory development, template analysis, framework, definition, interview study

## Abstract

Organizational purpose has flourished in the professional management literature, yet despite increased scholarly interest, academic knowledge and empirical research on the topic remain scarce. Moreover, studies that have been conducted contain important oversights including the lack of a clear conceptualization and misinterpretations that hinder the further development and understanding of organizational purpose. In view of these shortcomings, our interview study aimed to contribute to academic and societal conversations on the contemporary meaning and function of organizational purpose considering the voices and perspectives of 44 global experts. Employing template analysis, we defined organizational purpose as “an organization’s reason for being characterized by significance, aspiration, direction, unification, and motivation.” Moreover, we proposed an explanatory conceptual model, including drivers and outcomes of purpose, important boundary conditions, and explanatory mechanisms. Drawing on self-determination theory, person–organization fit theory, job characteristics theory, and conservation of resources theory, we were able to explain how and under what conditions these concepts are related to organizational purpose. In doing so, our research contributes to advancing the knowledge and understanding of organizational purpose and its effects on human lives within and outside organizations. Our study thereby enhances the understanding of the role of organizations in society and helps in evaluating whether organizations take responsibility by living their purpose in the society they are part of. As such, our study provides important insights for theory development, scale development, and further empirical research on organizational purpose and its effects in different streams such as OB, HRM, marketing, leadership, and strategy.

## Introduction

The paradigm of shareholder capitalism and limitless growth that equates purpose to profit or maximizing shareholder value (Friedman, 1970) has propelled humanity with great progress and prosperity. It also has led to undesirable economic, environmental, and societal externalities, such as wealth inequality, financial crises, accounting scandals, depletion of planetary resources, and climate change (Hurth et al., 2018; Peele et al., 2019; Williams, 2019). The associated declining confidence and trust in businesses and organizations (Hollensbe et al., 2014; Montgomery, 2019) call for change, particularly in Volatile, Uncertain, Complex, and Ambiguous (VUCA) markets (e.g., Mann and Harter, 2016). In response to this call, organizations have started to embrace a “revolutionary” paradigm for organizational purpose other than profit-making that acknowledges the interdependency of organizations, businesses, and society (Hollensbe et al., 2014; Harrison et al., 2019; Montgomery, 2019; Mañas-Viniegra et al., 2020). To determine how organizations actually take societal responsibility and act accordingly, a clear conceptual understanding of the contemporary meaning of organizational purpose is needed (Duska, 1997; Mañas-Viniegra et al., 2020).

In recent surveys (e.g., British Academy, 2019; McKinsey, 2020), professional management literature (e.g., Montgomery, 2019), and practitioner publications (e.g., Peele et al., 2019), the notion of organizational purpose has gained attention and traction, as well as organizational purpose-related concepts such as purpose-driven leadership, purpose internalization (i.e., the integration of organizational members’ personal beliefs and motivation with the organization’s purpose) (Rey et al., 2019), and personal purpose (i.e., individual purpose based on values, life goals, and the meaning attached to life), role purpose (i.e., peoples’ life purpose defined in terms of the work that they do), and societal purpose (i.e., a society with high levels of collectivism that share goals which governments, institutions, organizations, and individuals collaborate to achieve) (Haski-Leventhal, 2020). Despite increased scholarly interest (e.g., Hollensbe et al., 2014), academic research on organizational purpose remains scarce (Kempster et al., 2011; van Tuin et al., 2020; van Ingen et al., 2021) and mainly takes a management (e.g., Hurth et al., 2018) or employee perspective (e.g., van Tuin et al., 2020; van Ingen et al., 2021). Such perspectives focus on outcomes such as performance (e.g., Thakor and Quinn, 2013; Henderson and Van den Steen, 2015; Hurth et al., 2018; Gartenberg et al., 2019), attention and emotional intensity (Mañas-Viniegra et al., 2020), and work engagement (van Tuin et al., 2020; van Ingen et al., 2021). The conceptualizations in these perspectives, however, are not based on thorough academic research (Keller, 2015; Mañas-Viniegra et al., 2020) or focus on defining the concept solely in terms of “beyond profit maximization” (Thakor and Quinn, 2013; Henderson and Van den Steen, 2015). Moreover, organizational purpose is often confused with motives of top management to adopt purpose, referring to its consequences, such as financial performance (Hurth et al., 2018), which can be viewed as a tautology trap (MacKenzie et al., 2011). Furthermore, organizational purpose is often conflated with the concept of organizational purpose statement. Although these concepts are related, they are not the same. As our study suggests, an organizational purpose is the organization’s reason for being; a statement is a tool for communicating and articulating purpose in a concise and inspirational way. A statement consists typically of one or two sentences supposed to convey how the organization fills human needs or solves human problems and may impact the way how people perceive purpose (Collins and Porras, 2008; Alegre et al., 2018), but this is beyond the scope of our study. Operationalizations in the abovementioned perspectives are also limited. For example, organizational purpose is based on a professional and practical definition (van Ingen et al., 2021), or on items constructed from related though distinct constructs, such as meaningful work (Gartenberg et al., 2019), or on mission (i.e., what you want to achieve in terms of specific activities, specific goals, and a specific timeline), vision (i.e., an imagined future state of what it will be like when the purpose is being lived and the mission accomplished), and (shared) values (i.e., the way *how* you do business and not *why*) (van Tuin et al., 2020).

The lack of clear terminologies and a uniform definition that fits the present Zeitgeist (Montgomery, 2019; Williams, 2019; Mañas-Viniegra et al., 2020), multiple meanings (Hirsch and Levin, 1999), and misinterpretations (Oswald, 2019) of organizational purpose have occurred, which hinder theory (Hirsch and Levin, 1999; MacKenzie et al., 2011; Singleton, 2014) and scale development (MacKenzie et al., 2011). In view of these oversights in the existing literature, the present interview study aims to enhance the academic and societal conversations on organizational purpose through the meaning-making of experts, thereby providing a definition and an explanatory conceptual model including drivers of purpose and aspects influencing the effects of purpose on outcomes. The present study aims to make several contributions that allow developing an instrument to measure organizational purpose and test hypotheses concerning this concept. First, it enhances the understanding of organizational purpose by providing a historical background and by considering how experts from academia and practice (*N* = 44) (Galuppo et al., 2020; Otto et al., 2020) give meaning to organizational purpose and why it has gained more attention in recent years. Based on that understanding, we define organizational purpose as *an organization’s reason for being characterized by significance, aspiration, direction, unification, and motivation*. Second, drawing on Whetten (1989), we propose a conceptual framework that can form the basis for future research on organizational purpose. More specifically, we suggest that *human needs*, *human problems*, as well as *founding values* can be viewed as antecedents of organizational purpose. We distinguish three explanatory mechanisms of why organizational purpose affects outcomes, namely, *meaningfulness* (Hackman and Oldham, 1976; Lysova et al., 2019), *need fulfillment* (Hobfoll, 1989; Deci and Ryan, 2000), and *person-organization fit* (Kristof, 1996; Leiter and Maslach, 2003; Ryan and Deci, 2019). Third and final, by explaining how organizational purpose functions and what outcomes may be achieved at intra- and extra-organizational levels and within society, we also contribute to a relevant societal conversation. More specifically, the present study reduces confusion and conflation of organizational purpose with related constructs such as mission, vision, and (shared) values (Montgomery, 2019; Peele et al., 2019), and the concept of organizational purpose statement as a tool for communicating purpose (Collins and Porras, 2008; Alegre et al., 2018). It also reduces the odds of “purpose-washing” (Oswald, 2019, p. 28), i.e., the use of purpose as a misleading tool for profit-making, and the narrow view of organizational purpose as do-gooding for society (Fischer et al., 2019; Mañas-Viniegra et al., 2020).

Next, we provide a brief historical background that clarifies some of the contemporary confusion on organizational purpose, followed by the study’s methodology, results, discussion, and conclusions, including a roadmap for future research.

## Theoretical Background

### Historical Background

Throughout the past century, the concept of organizational purpose has been subject to dichotomous meanings and interpretations, pendulating back and forth depending on its Zeitgeist (Singleton, 2014). On the one hand, the meaning of organizational purpose has been seen as instrumental, objective, functional, and outward focused and synonymously used with words such as end, aim, goal, or objective (Drucker, 1954; Friedman, 1970; Jensen, 2002). On the other hand, its meaning has also been spiritual, telic, subjective, moral, ideal, emotional, and inward focused (Bartlett and Ghoshal, 1994; Springett, 2004). As the teleological, spiritual, or religious connotation of organizational purpose had led academics to weaken the legitimacy of the use of the concept of organizational purpose in organizational scholarship (Moore and Lewis, 1953; Simon, 1964), scholars applied substitutes for purpose with a broader scientific acceptance, such as objective and goal (Selznick, 1943; Drucker, 1954). For example, with objective in mind, Drucker (1954) defined organizational purpose as “to create a customer” (p. 37). Strikingly, the term objective led to a more quantifiable measure, such as profit maximization (Friedman, 1970), instead of the function that purpose used to encompass, leading to confusion about profit being the purpose of the organization. As a consequence, the concept of organizational purpose has suffered from a lack of importance for and attention by academics throughout the century and the dichotomous nature of purpose led to a tension in using the term that is still present in contemporary times (Singleton, 2014).

### Revival

In the 1990s, several attempts were made to revive the spiritual meaning of the concept of organizational purpose. For example, Collins and Porras (1991) defined organizational purpose as “an outgrowth of the organization’s core values and beliefs” (p. 38) and Bartlett and Ghoshal (1994) defined purpose as “the embodiment of an organization’s recognition that its relationships with its diverse stakeholders are interdependent” (p. 88). In short, the functional perspective shifted back to a more moral and ethical perspective (Bartlett and Ghoshal, 1994).

At the turn of the century, however, organizational purpose still regained little traction in academia (Basu, 1999; Springett, 2004) and practice (Ellsworth, 2002; Mourkogiannis, 2006) and is often defined as the overriding or fundamental reason for existing that drives strategy (Ellsworth, 2002; Springett, 2004). Not only did organizational purpose suffer from the ongoing debate between shareholder primacy and stakeholder theory (Jensen, 2002), but also the conflation and confusion with corporate social responsibility (CSR) and shared value hindered its revival (Porter and Kramer, 2011). For example, CSR focuses on social activities beyond the organization’s core activities related to its purpose. As such, the conflation and confusion of organizational purpose with CSR became the source of an inflated focus on social purpose as do-gooding with large impact on achieving sustainable development goals (Peele et al., 2019).

In the first decades of this century, the reevaluation of organizational purpose revived and recently, the view on purpose shifted from the organization to the role of the organization in society (e.g., Hollensbe et al., 2014). Themes emerged, such as meaningfulness, transcendence, and contribution to society, in terms of solving societal problems which can be seen as aspirational. For example, Hurth et al. (2018) defined organizational purpose as “an organization’s meaningful and enduring reason to exist that aligns with long-term financial performance, provides a clear context for daily decision making, and unifies and motivates relevant stakeholders” (p. 4). Also, Keller (2015) defined organizational purpose as “an aspirational reason for being which inspires and provides a call to action for an organization and its partners and stakeholders and provides benefit to local and global society” (p. 1).

The above exposé shows that a clear, concise, and uniform definition of organizational purpose is missing, but that some themes or characteristics are recurring, e.g., reason for being, unifying principle, and motivational. Table 1 shows a non-exhaustive overview of various definitions of organizational purpose and related recurring themes or characteristics throughout the 20th and 21st centuries.

**TABLE 1:** A non-exhaustive overview of recurring themes and definitions of organizational purpose.

Themes/characteristics	Definitions of organizational purpose	References
Unifying principle, direction, objective	“The objective of cooperation, that enables decision-making by giving meaning to the circumstances, and provides a vision of future possibility that serves as a unifying principle and has a role in coordinating individual efforts within the overall organizational system”	Barnard, 1938, p. 86
Motivational, direction, unifying principle	“Organizational purpose is the motivating force moving, guiding, and delivering the organization to a perceived goal. It is the driving force, the fuel, the bond, the intangible link that pulls the organization together to achieve success”	Reyes and Kleiner, 1990, p. 51
Reason for being	“The ultimate priority of the organization, its reason for existence, its raison d’etre”	Basu, 1999, p. 8
Social benefit, unifying principle	“.something that is perceived as producing a social benefit over and above the tangible pecuniary payoff that is shared by the principal and the agent”	Thakor and Quinn, 2013, p. 2
Reason for being, direction	“The reason for which business is created or exists, its meaning and direction”	Hollensbe et al., 2014, p. 1228
Aspiration, reason for being, motivational, benefit	“An aspirational reason for being which inspires and provides a call to action for an organization and its partners and stakeholders and provides benefit to local and global society”	Keller, 2015, p. 1
Meaningful, enduring, reason for being, guiding, unifying principle, motivational	“An organization’s meaningful and enduring reason to exist that aligns with long-term financial performance, provides a clear context for daily decision making, and unifies and motivates relevant stakeholders”	Hurth et al., 2018, p. 4

## Materials and Methods

### Sample

To reveal the rekindled debate on organizational purpose, we collected the voices and views of 44 experts (Otto et al., 2020) of whom 12 were academics and 32 were practitioners from a wide range of countries and backgrounds. These experts, via their professions and professional communities, are highly influential regarding the meaning-making process of “revolutionary” organizational purpose. We selected participants based on their recent research or publications on the topic of organizational purpose beyond profit-making in purpose-related fields, such as strategy, meaningful work, leadership, marketing, HRM, economics, and corporate social responsibility. We used keywords such as purpose, organizational purpose, corporate purpose, and business purpose to search for books, articles, and blogs. To obtain a broad view, we aimed for academics and practitioners working in and for organizations in different Western countries (e.g., United States or Germany), different industries (e.g., food and agriculture or professional services), and different business or research streams (e.g., strategy or marketing), and with different roles (e.g., C-level or employee) and job titles (e.g., founder or director). Specifically, those individuals who founded or cofounded an organization were relevant to be interviewed as they were expected to be able to explain why their company was founded and with what purpose. Table 2 shows the interviewee characteristics.

**TABLE 2:** Interviewee characteristics.

Nr	Type	Stream	Gender	Region	Role	Job title	Industry
1	Practitioner	Marketing	Male	UK	C-level	Co-founder	Professional services: management consultancy
2	Practitioner	Economics	Male	NL	C-level	Co-founder	Professional services: training and development
3	Practitioner	Marketing	Male	NL	Employee	Marketeer	Professional services: marketing and communication
4	Practitioner	Leadership	Male	NL	Entrepreneur	Founder	Retail
5	Practitioner	Marketing	Female	NL	C-level	Co-Founder	Professional services: marketing and communication
6	Academics	Economics	Male	NL	Researcher/lecturer	Researcher/Ph.D.	Research and Education
7	Academics	Leadership	Male	NL	Ph.D. candidate	Ph.D. candidate	Research and Education
8	Practitioner	HRM	Male	NL	Entrepreneur	Founder	Financial services industry
9	Practitioner	Strategy	Male	NL	Senior manager	Director	Information technology: software
10	Academics	Strategy	Male	NL	Researcher/lecturer	Researcher/Ph.D.	Research and Education
11	Practitioner	Leadership	Male	UK	Entrepreneur	Founder	Professional services: management consultancy
12	Practitioner	HRM	Male	FR	C-level	Founder/CEO	Entertainment and recreation
13	Practitioner	HRM	Female	AU	Senior manager	Director	Creative and cultural
14	Practitioner	HRM	Female	NL	Employee	HR manager	Professional services: human resources
15	Practitioner	Economics	Male	NL	Senior manager	Management consultant	Professional services: management consultancy
16	Practitioner	Leadership	Male	NL	C-level	Co-founder	Creative and cultural
17	Practitioner	Leadership	Male	NL	C-level	Founder/CEO	Entertainment and Recreation
18	Academics	Leadership	Female	US	Researcher/lecturer	Researcher/Ph.D.	Research and Education
19	Academics	Marketing	Female	CA	Researcher/lecturer	Researcher/Ph.D.	Research and Education
20	Practitioner	HRM	Female	AU	C-level	Co-founder	Information technology: software
21	Academics	Strategy	Female	AU	Professor	Professor	Research and Education
22	Practitioner	Leadership	Female	NZ	C-level	Founder/CEO	Professional services: training and development
23	Practitioner	Leadership	Male	NZ	C-level	Co-founder	Financial services industry
24	Academics	HRM	Female	AU	Professor	Professor	Research and Education
25	Academics	Marketing	Female	DE	Researcher/lecturer	Researcher/Ph.D.	Research and Education
26	Academics	Strategy	Male	UK	Researcher/lecturer	Researcher/Ph.D.	Research and Education
27	Practitioner	Leadership	Male	US	C-level	Founder/CEO	Professional services: training and development
28	Academics	HRM	Male	US	Researcher/lecturer	Researcher/Ph.D.	Research and Education
29	Practitioner	Marketing	Female	US	C-level	Founder/CEO	Wholesale
30	Practitioner	HRM	Male	NL	Senior Manager	Director	Retail
31	Practitioner	Marketing	Male	NL	C-level	Co-founder	Information Technology: software
32	Academics	Leadership	Male	SA	Professor	Professor	Research and Education
33	Practitioner	HRM	Female	IS	Employee	HR Consultant	Hospitality
34	Practitioner	Strategy	Female	AU	Senior manager	Director	Professional services: training and development
35	Practitioner	Leadership	Male	NL	C-level	Co-founder	Information technology: software
36	Practitioner	Leadership	Male	US	Senior manager	Vice President	Professional services: training and development
37	Practitioner	Marketing	Male	NL	C-level	Founder/CEO	Professional services: education
38	Practitioner	Leadership	Female	NL	C-level	Co-founder	Healthcare
39	Practitioner	Strategy	Male	NL	C-level	Co-founder	Information technology: software
40	Practitioner	Strategy	Male	NL	C-level	Founder/CEO	Professional services: management consultancy
41	Practitioner	Economics	Male	NL	Senior manager	Services leader accountancy	Food and agriculture
42	Academics	Economics	Male	DE	Researcher/lecturer	Researcher/Ph.D.	Research and Education
43	Practitioner	HRM	Female	US	C-level	Founder/CEO	Professional services: education
44	Practitioner	Strategy	Male	NL	Employee	Logistics strategist	Logistics and supply chain

### Data Collection and Analysis

To analyze the interviews, we used template analysis (King, 2004) to build on previous work but to avoid constraining the analysis to established findings (Crabtree and Miller, 1992). We collected data through 44 individual semistructured interviews that lasted between 22 and 77 min, with an average duration of 35 min. All interviews were audio-recorded and transcribed verbatim. Data were collected using a semistructured interview protocol for comparison across interviewees with the goal to obtain a better understanding of organizational purpose. Examples of interview questions are “could you please describe any theme, idea or concept that comes to mind when you think of organizational purpose?” and “how would you define organizational purpose?” Thus, we specifically asked for the idea or concept of organizational purpose and not for the organizational purpose statements of organizations. During the interviews, we also carefully asked additional exploratory follow-up and non-directive probing questions to ensure not to direct the interviewees toward the perspective of the researchers (King, 2004). Data were analyzed based on a set of *a priori* codes, which were then expanded upon as additional themes emerged from the data analysis (Crabtree and Miller, 1992).

### Coding

As our aim was to build theory, we began with the fundamental theoretical building blocks as the first layer higher-order *a priori* coding (Whetten, 1989), namely, what, how, why, and who/where/when. “What” refers to the variables, constructs, concepts, and characteristics of organizational purpose. “How” pertains to relationships and patterns among the “what” factors. Typically, causality is introduced between organizational purpose and other dependent and independent variables of interest (i.e., antecedents and consequences). “Why” refers to explaining the reason why organizational purpose is related to other variables and the assumptions about the underlying causal mechanisms. Finally, “who/where/when” represents the conditions that place limitations on the propositions that come forth of the theoretical model. We used the recurring themes and characteristics from existing literature (see Table 1) as *a priori* codes for “what,” specifically to come to a definition of organizational purpose.

### Final Template

**FIGURE 1:**
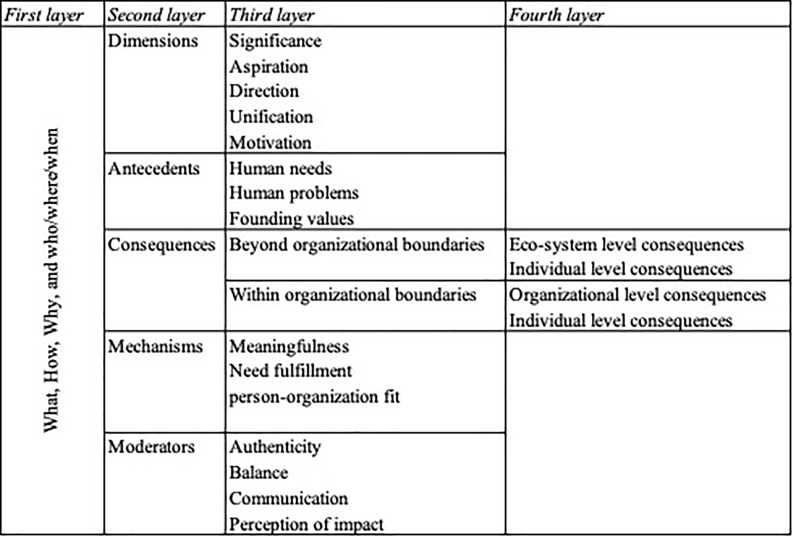
Final template: dimensions, antecedents, consequences, mechanisms, and moderators of organizational purpose.

The final template was developed through iteratively modifying and expanding the initial template based on *a priori* themes with newly identified emerging themes throughout the coding process (Crabtree and Miller, 1992; Randall et al., 2007). As such, we introduced second, third, and fourth layers of coding as can be seen in Figure 1, which shows our final template. For example, as second-layer subclasses of the first layer “how” category, we specified “antecedents” and “consequences.” As a third-layer coding, we identified variables such as “meaningfulness” and “need fulfillment” as subclasses of the second-layer “mechanisms.” Moreover, we identified individual-level variables, such as “engagement” and “commitment” as a fourth-layer coding of the third layer “within organizational boundaries” that is part of the second layer “consequences.”

After coding all interviews and comparing all coded transcripts, no new themes emerged from the data, suggesting that further interviews probably would not expand the template with new themes. Following Miles and Huberman (1994), we found agreement among two coders at the third- and fourth-layer coding to be 0.81, which exceeds the threshold of 0.80 (Miles and Huberman, 1994; O’Connor and Joffe, 2020). Reflexivity was important throughout development of the template to avoid researcher subjectivity (King and Brooks, 2017). Through conversation, questioning, and discussion, the authors agreed upon the layers of coding and which codes would be the final codes on each passage of text. Furthermore, all authors reconciled any disagreements or unclarities on the final coding through questioning and discussion, resulting in a final template of 19 third- and fourth-layer codes.

In addition to the process described above, we took another step to increase the credibility and confirmability of our study. We started a secondary coding process to determine if the final template fit the data (King and Brooks, 2017). Following previous studies using template analysis (e.g., Randall et al., 2007; Kidd, 2008), we gave three doctoral students in organizational behavior and HRM with experience in qualitative research and who were not familiar with the study our final template along with 63 passages of text in random order, preventing any pattern recognition (for each code, three passages of text were selected from random interviews). We asked them to indicate which code best reflected the textual passage. The percentage of agreement was 0.81, which exceeds the threshold of 0.80 (Miles and Huberman, 1994; O’Connor and Joffe, 2020).

### Developing a Conceptual Definition of Organizational Purpose

We followed MacKenzie et al. (2011) in developing a conceptual definition of organizational purpose. We examined prior literature on related constructs, we specified the nature of organizational purpose’s conceptual domain, and we specified the conceptual theme that encompasses a description of the characteristics, dimensionality, and stability of the construct.

## Results

In this section, following the building blocks of a theory (Whetten, 1989), we explore the dimensions, antecedents, consequences, mechanisms, and moderators relating to organizational purpose, through analyzing how experts view and talk about the concept.

**FIGURE 2:**
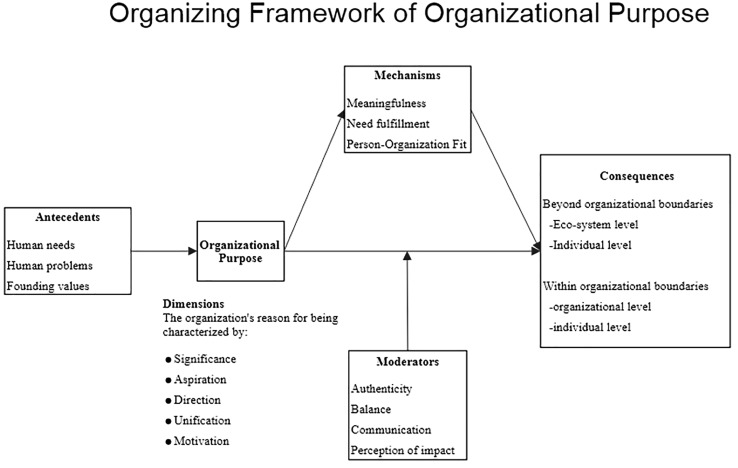
Organizing framework of organizational purpose.

Based on our analysis, organizational purpose can be defined as “*an organization’s reason for being characterized by significance, aspiration, direction, unification, and motivation*.” The characteristics can be considered as distinguishable facets, and omitting any of them would restrict the conceptual domain of organizational purpose in an important way. Following MacKenzie et al. (2011), we thus argue organizational purpose to be multidimensional and the subdimensions are viewed as defining characteristics. The conceptual model derived from our data analysis (Figure 2) presents five dimensions of organizational purpose, namely, significance, aspiration, direction, unification, and motivation. Moreover, human needs, human problems, and founding values are depicted as antecedents of organizational purpose. Based on the analysis of our interview data, we propose that these antecedents act as causal links to organizational purpose. This needs to be further examined in quantitative research. Two types of consequences can be distinguished, namely, consequences beyond organizational boundaries (at the ecosystem level and the individual level) and within organizational boundaries (at the organizational level and individual level). Furthermore, the model presents three explaining mechanisms (meaningfulness, need fulfillment, and person–organization fit) and four moderators (authenticity, balance, communication, and perception of impact). To substantiate our findings, a selection of 19 excerpts exemplary for the meaning making and views of the experts that highlight the different aspects of our model is presented below and reflected upon. Table 3 presents additional interviewee excerpts which support our findings.

**TABLE 3:** Additional excerpts of interview data.

	Excerpts	
Dimensions	*Significance: “So, the purpose of a person is not just to breathe and to survive and the purpose of an organization is not just to maximize profit and to employ people or just achieve its goals. It’s about how they can use the organizational power, resources and talent to also benefit society and the community in which it operates*.” (#*21, academic, strategy, female, AU).*	*Aspiration: “It is about what you aspire to create, meaningfulness in the world and in your life*.” (#*19, academic, marketing, female, US).*
	*Unification: “A purpose has a very, how would you call it? It has a unifying consequence. Like oftentimes when things fall apart, it’s because we don’t have that clarity about one’s purpose. If an organization doesn’t have a sense of purpose, you have that risk that personal purposes tend to have a higher priority. Having a collective purpose brings people together*.” (#*15, practitioner, economics, male, NL).*	*Motivation: “Your people will be so motivated to work that you don’t need to manage them. They love to be there because they want to come to do it. We give them a lot of energy.*” (#*35, practitioner, leadership, male, NL).*
	*Direction: “So, a very good purpose would enable to know where it is going, what’s the direction, in which it is leading everyone. So, a purpose-driven organization inspires and provides a shared narrative that connects people and provides them with a sense of meaningfulness*.” (#*21, academic, strategy, female, AU).*	
Antecedents	*Human needs: “Oftentimes when we think about purpose, we sometimes also call it like one’s calling. You see something is calling to you from the outside. Like it’s not that you come up with something and then, this is what I want to do, but it’s what is really needed in society*.” (#*15, practitioner, economics, male, NL).*	*Human problems: “We are in business to save the home planet*.” (#*7, academic, leadership, male, NL).*
	*Founding values: “But the purpose that you know starts with something that the company stands for and that’s something that usually the founders of the company have to decide*.” (#*19, academics, marketing, female, CA).*	
Consequences: Beyond organizational boundaries	*Eco-system level consequences: “The purpose of an organization for me is to is to contribute to the health of the higher-level systems that it operates in. You know, it’s about self-transcendence. It’s about the fact that an organization is always a part of various systems*.” (#*26, academic, strategy, male, UK).*	*Individual level consequences: “And of course, customer loyalty may be like being less vulnerable to market changes in the marketplace*.” (#*20, practitioner, HRM, female, AU).*
Consequences: Within organizational boundaries	*Organizational level consequences: “The companies which are truly purpose driven they have better financial results*.” (#*4, practitioner, leadership, male, NL).*	*Individual level consequences: “But what I’ve been discovering is that it really helps with employee engagement. and it helps with employee retention*.” (#*33, practitioner, HRM, female, IS).*
Mechanisms	*Meaningfulness: “If your purpose is not meaningful for me. It’s not a purpose.*… *So, I think every purpose should be meaningful.*… *It doesn’t have to be meaningful*… *what’s meaningful for you, doesn’t have to be meaningful for me. But in the essence, I think if your purpose is benefiting the planet or humanity, it is, it will be meaningful*.” (#*35, practitioner, leadership, male, NL).*	*Need fulfillment: “I think at the heart of purpose is human connection, the human need to belong to something greater than ourselves, bigger than who we are on our own*.” (#*13, practitioner, HRM, female, AU).*
	*Person-organization fit: “When you allow people and enable them to live by their personal purpose but also creating alignment between the personal and the organizational purpose and even maybe societal purpose, you create a real sense of meaningfulness. when there is a disconnect, people are not engaged. My other concern around purpose is the disconnect between those different levels of purpose*.” (#*21, academic, strategy, female, AU).*	
Moderators	*Authenticity: “My biggest concern is that a lot of organizations, don’t really understand what it means to be purposeful and so they might try to as a touchy-feely story. Instead of really, seeing how they can feel holistic about the purpose embedding the purpose in every aspect of the organizational life and enabling people to lead a purposeful life. And to lead by the purpose, I don’t want it to be empty words, I want it to be something that everyone feels*.” (#*21, academic, strategy, female, AU).*	*Balance: “Now a lot of people think that purpose is about like transcending the ego, like not being ego driven. And that’s actually not true, because your purpose needs your ego*…. *Your purpose, like, no, you got to reconcile it with your ego structure*.” (#*36, practitioner, leadership, male, US).*
	*Perception of impact: “As long as you have a purpose, so you know where you’re trying to head. And you have a way of understanding whether you are heading in the right direction. So, you have a way of measuring progress. You need to be able to measure that*.” (#*26, academic, strategy, male, UK).*	*Communication: “Those organizations that think, act, and communicate on why it is what they do, the high purpose, the cause, their belief, they open up the possibility inspiring trust and loyalty.*… *When you can think, act, and communicate clearly starting with why, you give the people who are listening the opportunity to decide whether they believe that too, and if they do, they will choose to buy what it is you have*.” (#*1, practitioner, marketing, male, UK).*

### Dimensions of Organizational Purpose

#### Significance

In response to the question regarding what organizational purpose means to them, the interviewees stressed purpose to have a positive impact or contribution on the lives of both employees and people outside the organization. Interviewees suggested that a positive contribution needs to be related to solving human problems or fulfilling human needs. As such, the interviewees explained that purpose can be perceived good or bad, it is subjective as people experience different needs and problems. In view of this suggestion, the interviewees mentioned the important contributing role each organization has in the society it is part of and that purpose has both an immanent and transcendent aspect relating to *significance*. Such a contribution can be large, for example, ending world hunger, or small. One interviewee mentioned an illuminating example of the importance of a car wash:

“No one wakes up and wants to work at a car wash. So, we employ people who don’t have a high school degree. He said that we are people’s education that they never had. And so, he realized that while we wash cars, our purpose has to be to prepare these people for their future. And then, the second part of their purpose came about when we asked: “Well, why do people get their cars washed?” And so, he went out there and asked people why they wash their cars, and it was things like: I’m getting my car washed, so that I can drop my kid off at college as he is the first in our family to go to college. I want my car to look nice, going to a funeral, going to a graduation. Just part of the everyday routine to make you feel good and he quickly realized those two things, preparing people for the future, their people, and then providing for the community” (#28, academic, HRM, male, US).

We define significance as “the degree to which the organization has a substantial positive contribution to or impact on the lives or work of people, whether within the organization or in the external environment outside the organization, such as local or global society.” According to this definition, purpose is not a distinctive factor, since different organizations can have the same purpose. Moreover, purpose is not only significant to the organization itself and its shareholders (Jensen, 2002), nor is purpose solely focused on doing good to others (Fischer et al., 2019; Mañas-Viniegra et al., 2020). We conclude that purpose is subjective, and its significance and contribution depend on whose needs are fulfilled or whose problems are solved.

#### Aspiration

Interviewees indicated that they associate purpose with the hope or ambition of achieving “something” in the future that is worthwhile and significant that people pursue for its own sake. That “something” is significant as it is related to need fulfillment, or the solving of problems. It is strongly desired but difficult or maybe impossible to achieve. As such, it involves a connection between present and future. The interviewees explained that aspiration can be viewed as a higher transcendent potential that is enduring over time, something that one must continually strive for and is challenging rather than some end, mission, or goal that can be accomplished. For instance, a practitioner in HRM gave the example of ending world hunger, which is a need that cannot be fulfilled:

“So, when you look at the sustainability development goals from the UN [United Nations, authors], they are really big and lofty. Something to aspire in the long term. I mean, you cannot end world hunger, but every action you take, you do something in the short term that contributes to your purpose.” (#33, practitioner, HRM, female, IS).

The interviews suggest that aspiration can be defined as “the hope or ambition of achieving the fulfillment of human needs in the future (i.e., significance), strongly desired yet difficult or maybe impossible to achieve, that one must continually strive for.” As such, aspiration differs from vision, i.e., an imagined future state of what it will be like when the purpose is being lived and the mission(s) accomplished (Collins and Porras, 2008). Moreover, aspiration differs from concrete, reachable organizational goals (Drucker, 1954). We conclude that aspiration relates to the continuous striving for fulfilling recurring or continuous needs or problems. As such, it has no end state and aspiration differs from concepts such as vision and goals.

#### Direction

The interviewees described direction as the path or course to fulfilling the needs or solving the problems that are described in the significant and aspirational aspects of purpose. They also indicated that the directional aspect of purpose guides decision-making promotes goal orientation and provides order and coherence of actions. Some interviewees specifically addressed the importance of the directional aspect of purpose in relation to the highly VUCA world we live in. In addition, they remarked that purpose creates and organizes the basis for developing strategic goals and both higher- and lower-order organizational goals in many different domains. One interviewee, a practitioner in marketing, coined the term “north star” for the directional and guiding aspect of purpose:

“It’s sort of this like kind of like a ‘north star’ for your business.” (#5, practitioner, marketing, female, NL).

Based on the above analysis, direction can be defined as “the path or course to fulfilling the significant and aspirational aspects of purpose, thereby guiding decision-making, promoting goal-orientation, and providing order and coherence of actions.” This definition is in line with previous research on the directional aspect of purpose (e.g., Barnard, 1938; Bartlett and Ghoshal, 1994).

#### Unification

The interviewees addressed the societal problem of disconnection in our current VUCA world, stressing that purpose is necessary for reconnecting and providing shared understanding and meaning. As such, the unification aspect of purpose can play an important role in building human connections among people with a shared purpose. More specifically, organizational purpose can unify, bind, and connect people inside and outside organizational boundaries. Through the significant, aspirational, and directional aspects, organizational purpose has the potential to promote collaboration and as such may provide a sense of belongingness, or relatedness at the emotional level. The interviewees also indicated that purpose has the capacity to promote cooperation between the organization and stakeholders. A researcher in HRM explained the connection at the emotional level of purpose with individuals:

“An organization that’s got a really clear and inspiring purpose connects with you on an emotional level.” (#24, academic, HRM, female, AU).

Based on our analysis, we define unification as “the connecting or binding of people to the organization and its purpose, through shared understanding of the significant, aspirational, and directional aspects of purpose, thereby fostering belongingness, relatedness, and connectedness at the emotional level, and collaboration (inside the organization) and cooperation (outside the organization).” This understanding is in line with previous research on the unification aspect of purpose (e.g., Hurth et al., 2018).

#### Motivation

The interviewees vividly described that they view organizational purpose as a motivational force that is energizing, inspirational, and action oriented. They considered organizational purpose literally as a valued source of energy, a force that sets people within and outside the organization at all levels in motion, that drives action, and that pulls into the future. One interviewee gave this example that even the employee with a job in which meaningfulness of the organization to society cannot be directly experienced (e.g., a staff or support function of the organization) may experience being motivated through the organization’s purpose:

“When your company has a higher purpose, then it’s very motivating and engaging for your employees. You know, even employees that don’t have very exciting jobs, if they know that they are part of alleviating world hunger that’s going to make their boring job a little bit more motivating.” (#18, academic, leadership, female, US).

Our analysis revealed that motivation can be defined as “the energization of voluntary activities or behaviors either done for their inherent interest (i.e., need fulfillment) or done for the reason of fulfilling the organization’s significant, aspirational, directional and unification aspects of purpose.” As such, motivation can be characterized by high levels of energy, inspiration, intensity (effort), and persistence (duration) of voluntary (volitional) action and applies to people inside and outside the organization. This characterization is in line with previous research on the motivational aspect of purpose (e.g., Reyes and Kleiner, 1990; Hurth et al., 2018).

### Antecedents

#### Human Needs

The interviewees considered organizations as a part of society that can serve and advance society by fulfilling human needs or broader societal needs to foster well-being. They explained that human needs are a broad concept, are context dependent, and can be approached through different stakeholder lenses (society, environment, economy, and people). A researcher in marketing explained that all organizations have a purpose and that purpose is need fulfilling:

“But when you look at purpose as the reason for which something is done or created or for which it exists. That definition really opens it up to any organization if it exists, has a human bettering, need fulfilling purpose” (#25, academic, marketing, female, DE).

Our interpretation of the data is that people organize to fulfill human needs effectively and efficiently (Barnard, 1938; Collins and Porras, 2008). As such, every organization can be taken to have a purpose that fulfils one or more human needs.

#### Human Problems

The interviewees also explained that the purpose of organizations needs to serve and advance society by solving societal problems to foster well-being. However, problems can be approached through different perspectives. From a societal perspective, for example, the interviewees mentioned not only problems such as racism, poverty, crime, and wealth inequality but also the disconnection of society through the decay of religion and communities or the injustice people are facing. From an environmental perspective, they indicated problems such as climate change, pollution, and scarcity of water and food. From an economic perspective, interviewees brought forward problems such as depletion of resources, overconsumption, and disengagement at work. A practitioner in leadership phrased the problem-solving focus of purpose as:

“This whole idea of purpose that it’s the contribution you make, the human problem you exist to solve.” (#4, practitioner, leadership, male, NL).

Our analysis showed that different types of human problems can be viewed as predictors of organizational purpose (Barnard, 1938; Collins and Porras, 2008). Solving problems is distinct from but closely related to fulfilling needs, whether these are our own needs or someone else’s needs.

#### Founding Values

The interviewees explained the importance of founding values in relation to purpose, as what is valued often translates to fulfilling needs or solving problems. Founding values were shown to correspond with the founders’ values, beliefs, ideals, and aspirations. As a professor in strategy remarked:

“Companies were actually founded, not to sell products, they were founded to address an issue, that the founder found very compelling.” (#21, academic, strategy, female, AU).

The above analysis shows a difference between founding values and organizational values, a finding that has not been established in previous research (e.g., Collins and Porras, 2008). Founding values relate to fulfilling of needs or solving problems. By contrast, organizational values have an important role in guiding behavior, for example, the values of honesty or transparency. We believe that organizational values might change over time and are likely to be cultural, Zeitgeist, and context dependent. We observed a clear relationship between what is valued and needed and what are considered to be shared values between an organization and the ecosystem it is part of. We conclude that the founding values intrinsic to purpose cannot be violated without affecting purpose (Modesti et al., 2020).

### Consequences Beyond and Within Organizational Boundaries

#### Consequences Beyond Organizational Boundaries

The interviewees indicated the distinction between consequences at the ecosystem level the organization is part of and consequences at the individual level. With regard to ecosystem-level consequences, interviewees referred to the consequences of organizational purpose for society, its constituents, and thus the ecosystem it is part of. They explained that these consequences boil down to having a positive impact on well-being, quality of life, flourishment, and advancement of humanity for generations to come. Some interviewees remarked that without purpose an organization will not have a license to operate. In that way, purpose was viewed as the legitimacy for doing activities to provide value for stakeholders (e.g., shareholders, customers, partners, and potential employees). A practitioner in leadership expressed purpose as well-being of humanity and the planet for generations to come:

“For me purpose is to increase the well-being of the planet and the people living on the planet for generations to come. Your purpose is benefiting the planet or humanity. So, your product or service is making this world a better place. If you are really purpose driven, then this world will become a better place.” (#35, practitioner, leadership, male, NL).

Our analysis showed that purpose clarifies the role of an organization in society (cf. Hollensbe et al., 2014), its legitimacy of society, and its constituents and may provide evaluation criteria whether an organization is on the right track (cf. Duska, 1997).

The interviewees also addressed the beneficial outcomes of purpose on individuals outside the organization. They explained that organizational purpose may positively affect individuals whose problems are solved or needs are fulfilled. At the individual level, the interviewees mentioned that living purpose likely leads among others to stakeholder engagement, attractiveness, loyalty, commitment, and trust:

“I think about values. I think about engagement. Well, a shared goal, loyalty and sense of commitment, sense of pride, consumer engagement, stakeholder engagement.” (#19, academic, marketing, female, CA).

Purpose thus can have positive effects on individuals whose needs are fulfilled, whose problems are solved, or by resonating with their individual purpose or values (cf. McKnight and Kashdan, 2009; Rainey, 2014). Our interpretation of the individual-level consequences outside the organization is explained in the next section, mechanisms.

#### Consequences Within Organizational Boundaries

The interviewees indicated the distinction between consequences at the organizational level and consequences at the individual level. With regard to organizational-level consequences, they indicated that living the organization’s purpose can lead to an increase in organizational performance, innovativeness, and resilience. Moreover, an overarching purpose enables leadership to set the mission, i.e., to set the short-term concrete achievable goals and objectives enabling leadership to derive the strategy, to guide decision-making, and to link these with the order and planning of activities. Interviewees also explain that purpose enables leaders to set the vision:

“The purpose is what drives everything in the organization. The mission, the vision, the strategic planning.” (#21, academic, strategy, female, AU).

Our interpretation of the data is that purpose enables leadership to define the mission, vision, and strategy of organizations (Collins and Porras, 2008). We contend that organizational-level outcomes such as performance, innovativeness, and resilience are influenced not only by the quality of objectives but also by individual-level consequences of purpose (Lysova et al., 2019).

The interviews also indicated that purpose could lead to meaningful work and engagement at the individual level. In line with previous research (van Tuin et al., 2020; van Ingen et al., 2021), some interviewees addressed benefits such as commitment, fulfillment, happiness, loyalty, optimism, pride, satisfaction, trust, a sense of belonging, and well-being:

“There are a lot of things you do in a company to improve the happiness of the people that work there. Having purpose is a really important one.” (#30, practitioner, HRM, male, NL).

Now that we have described the dimensions, antecedents, and consequences of organizational purpose, the next section explores the mechanisms explaining why purpose influences outcomes.

### Underlying Mechanisms

#### Meaningfulness

The interviewees mentioned that organizational purpose can be perceived as meaningful to individuals and that through meaningfulness positive outcomes can be reached. Whether purpose is meaningful, however, depends on the individual’s perception and is in the eye of the beholder. To engage purpose-driven people, you need to provide a sense of meaningfulness, guided by the organization’s purpose. As one interviewee remarked:

“So, a purpose-driven organization inspires and provides a shared narrative that connects people and provides them with a sense of meaningfulness. … And a purpose-driven organization engages people in a way that we’ve never seen before. You really want to engage people. We are purpose-driven animals, so if you want to engage people you need to give them a sense of meaningfulness.” (#21, academic, strategy, female, AU).

In the literature of meaningful work, the concept of meaningfulness is well described (e.g., Lysova et al., 2019). Thus, we believe the Job Characteristics Model (JCM; Hackman and Oldham, 1976) might be a relevant model to incorporate in organizational purpose theory as organizational purpose can be assumed to foster the perception of task significance, which triggers the psychological state of perceived meaningfulness that must be present for internally motivated work behavior and may therefore act as a mediator in the relationships between organizational purpose and work outcomes (Hackman and Oldham, 1976). By contrast, the meaningfulness of organizational purpose has not yet been investigated in relation to other stakeholders other than employees. Research in marketing indicates that meaningfulness is necessary in marketing for achieving outcomes (Lehnert et al., 2014; Zuo et al., 2019), as such organizational purpose may play an important role in marketing as a research stream.

#### Need Fulfillment

Interviewees explained that organizational purpose originates from human needs; thus, the fulfilling of these needs may lead to a positive state of mind or a positive feeling of the person whose need is fulfilled. One interviewee indicated, for example, the fulfillment of customer needs by a baker and a shoemaker:

“The baker bakes bread to fulfill the need of his customer to eat, the shoemaker makes shoes to fulfill the need for feet comfort and protection. By fulfilling these needs, customers experience satisfaction.” (#12, practitioner, HRM, male, FR).

Other interviewees explained that people are in need of meaningful work, experience significance in what they do for others, and are in need of belonging to something greater than themselves. The interviewees mentioned that organizational purpose can fulfill these psychological needs of people who work for an organization. According to the interviewees, (psychological) need fulfillment is an important mechanism that explains the effects of purpose on outcomes. The fulfillment of (psychological) needs is well described in motivational theories (e.g., Kanfer et al., 2017). We believe that self-determination theory (SDT) (Deci and Ryan, 2000) and conservation of resources (COR) theory (Hobfoll, 1989) might be relevant theories to incorporate in organizational purpose theory. Organizational purpose can be perceived as significant and valued in its own right, which may lead to the experience of a high degree of volition or willingness to act, and thus fulfils the need for autonomy. Furthermore, as the interviewees explained, the unifying power of purpose in fostering belongingness may lead to fulfillment of the need for relatedness. As such, SDT may explain the positive effects of purpose on outcomes in both an intra-organizational environment (e.g., Ryan and Deci, 2019) and an extra-organizational environment with, for example, effects on customers (Gilal et al., 2019). The interviewees indicated that organizational purpose is considered as a valued source of energy that sets us in motion. According to Hobfoll et al. (2018), among commonly valued resources are well-being, self-esteem, and a *sense of purpose*. We thus see a link to consider organizational purpose as an organizational resource to individuals inside and outside the organization. We believe that COR theory can be considered a motivational theory that explains the effects of purpose on outcomes by the need for conservation of resources. As such, purpose can be considered an organizational resource for employees (Hobfoll et al., 2018) and a customer resource in marketing processes (e.g., Smith, 2013; Hollebeek et al., 2019).

#### Person–Organization Fit

The interviews showed that the level of fit between an individual and the organization is a mechanism explaining the affects or organizational purpose on outcomes. Interviewees mention that purpose can be motivational if individuals perceive a fit between their own purpose, their team’s purpose, and the organization’s purpose:

“I found it incredibly personally motivating that I had such a clear alignment between my purpose, our team’s purpose and the greater organization’s purpose.” #24, academic, HRM, female, AU).

Besides the level of fit referring to purpose, interviewees also mentioned value fit or alignment of values between the individual and the organization and specifically the congruence between the individual’s values and the founding values (Grant, 2008). Perceived P–O fit may become a source of self-definition, so that individuals are more likely to be attracted to an organization when its purpose matches their own sense of who they are. The mechanism of person–organization (P–O) fit generally relates to the level of fit between an individual and the organization in terms of goal congruence and values fit (Kristof, 1996). The effect of P–O fit as a mediator in relationships between organizational variables and outcomes has been extensively researched in individuals in a work context (e.g., Leiter and Maslach, 2003; Grant, 2008). Furthermore, P–O fit has been touched upon in employer attractiveness (e.g., Klimkiewicz and Oltra, 2017) and marketing (e.g., Yaniv and Farkas, 2005). In addition, fit perceptions generate a sense of relatedness toward the organization, which supports the satisfaction of the basic psychological need belongingness in SDT and thus fosters autonomous motivation, which in turn positively affects outcomes (Deci and Ryan, 2000). As such, we can conclude that P–O fit theory, specifically the fit on purpose and founding values, can be seen as an explaining mechanism between purpose and outcomes in organizational purpose theory.

### Moderators

#### Authenticity

The interviewees were very clear about the importance and necessity of being authentic as an organization and as leaders of an organization. They indicated that close alignment between purpose, words, and behavior will enhance the effects of purpose on outcomes. A practitioner in leadership explained that this alignment should be clear in everyday practice:

“And it’s actually a very high standard of living. It’s not a marketing trick. It’s an extremely high standard to live up to as an organization. If you say you commit to this, you better do it because otherwise you can understand why people don’t trust you. … But then it has to be real, words and actions have to go hand in hand.” (#16, practitioner, leadership, male, NL).

We interpret authenticity as not only having purpose, but to be purposeful and to act and to live the purpose by example. This interpretation enables us to recognize “purpose-washing” (Oswald, 2019, p. 28), i.e., the use of purpose as a misleading tool for profit-making. Inauthentic organizational and individual behavior will lessen the effects of organizational purpose on outcomes. Inauthentic efforts can backfire, particularly when people perceive a large discrepancy between words and deeds (Bailey et al., 2017). Furthermore, inauthenticity might reveal Machiavellianism in organizations, i.e., personal motives relating to power and wealth gain the upper hand, eventually negatively affecting the organization and people (Belschak et al., 2018).

#### Balance

The interviewees mentioned that purpose at its core has significance to both people outside and people inside the organization and stressed the importance of balancing the transcendent aspect of purpose that focuses on others with the self-interest aspect that focuses on survival and continuity. In this vein, one interviewee mentioned the important role of balance:

“So, what is it that you as an organization are adding to the connected network? But also, what are you taking out and is there a balance in that transaction? Because if you pull out too much, there will be a disbalance in the system. So, for me, knowing your purpose is knowing who you are in the system that you are part of.” (#38, practitioner, leadership, female, NL).

Another relevant point of view to balance was coined by a few interviewees in which they explained the term “purpose paradox” as the harmony between what is good for others is good for the organization, meaning counterintuitively focusing on benefitting others leads to more profit. Interviewees explain that this can be difficult in times of economic downturn. We interpret balance in a way that egotism and altruism go hand in hand, drawing a parallel with individual purpose (Lips-Wiersma, 2002; Lips-Wiersma and Morris, 2009). Any disbalance will lead to either an unhealthy focus on profit, competitive advantage, or growth (e.g., Friedman, 1970; Jensen, 2002). Such a disbalance will be a high cost to the society the organization is part of or lead to an unhealthy focus on altruistic behavior (e.g., Sisodia et al., 2007; Keller, 2015) that in the end will likely threaten the organization’s continuity. Our interpretation of balance differs from the shared value concept (Porter and Kramer, 2011) that embraces the paradigm of continuous and greater growth for organizations and greater benefits for society. By contrast, our view of balance might in certain occasions lead to degrowth in maintaining healthy organizations and benefit for society. We believe that balancing egotistic and altruistic tensions benefits both the organization and the system it is part of.

#### Communication

Communicating a clear and concise purpose, consistently, frequently, and toward all stakeholders is something many interviewees find important to see the positive outcomes of purpose and that communicating your purpose fosters outcomes more than solely having purpose. Other interviewees stress the importance of communicating the purpose narrative to ensure people understanding the purpose and to enforce the power of purpose. As a researcher in strategy explains:

“I think it would also provide a narrative that connects people. So, if you have a really good purpose you also provide a story for the organization for people who want to understand. … So, a purpose-driven organization inspires and provides a shared narrative that connects people and provides them with a sense of meaningfulness.” (#21, academic, strategy, female, AU).

Our analysis showed that communication will likely affect the relationship between purpose and outcomes. However, communication does not indicate how purpose is being perceived, as the perception of good or bad lies within the eye of the beholder. Whether the formulated organizational purpose influences the individual-level psychological processes much depends on how purpose is perceived by individuals as intended organizational practices do not necessarily associate with individual perceptions (e.g., Nishii and Wright, 2007; Drucker and Maciariello, 2008). As such, communicating purpose in a clear and concise way plays an important role in the perception of purpose by individual stakeholders.

#### Perception of Impact

The interviewees mentioned that receiving feedback on the purpose’s impact and contribution or feedback in results of purpose on work can create higher levels of perceived meaningfulness and subsequently higher levels of positive outcomes. A practitioner in leadership gave an example of the importance of an organizational purpose’s significance by comparing a nurse in healthcare with a controller in financial services:

“There are also people who don’t really see what they are contributing to with their work. I think if you are a nurse, then you really see every day what your contributions are to society. But if you are doing something working, for example, for a bank and you’re just making spreadsheets and you have no clue what the impact of this spreadsheet is on the end customer or society than you do work that is not meaningful.” (#35, practitioner, leadership, male, NL).

We interpret the necessity of the reinforcing power of impact or feedback in terms of the embodiment of perceiving the contribution you make as an organization or as an individual onto others. This interpretation is in line with previous research (Grant, 2007, p. 399) where perceived impact on beneficiaries is defined as “the degree to which employees are aware that their actions affect others.”

## Discussion

This study aimed to contribute to academic and societal conversations on the meaning and function of organizational purpose considering the voices and views of experts (Galuppo et al., 2020; Otto et al., 2020). Although academic attention for organizational purpose has increased, scholarly research on this topic remains scarce (e.g., van Tuin et al., 2020; van Ingen et al., 2021) particularly in comparison to the attention organizational purpose has received in the professional management literature (e.g., Montgomery, 2019). More specifically, current studies have failed to theorize about organizational purpose, leading to a lack of a clear conceptualization (Williams, 2019; Mañas-Viniegra et al., 2020), multiple meanings (Hirsch and Levin, 1999), and misinterpretations (Oswald, 2019). To be more explicit, most studies conceptualized purpose not from an academic stance but from a practical one (e.g., Keller, 2015), in vague terms such as a concrete objective that reaches beyond profit maximization (e.g., Henderson and Van den Steen, 2015), or in terms of desired outcomes such as financial performance which are fueled by individual motives (Hurth et al., 2018). As a consequence, theory development and empirical research are hindered. Through 44 interviews with experts around the globe, we developed a better understanding of the contemporary meaning and function of organizational purpose that acknowledges the interdependency of organizations, businesses, and society (Hollensbe et al., 2014; Harrison et al., 2019). This understanding allowed us to contribute to academic and societal conversations in three ways. First, we introduced an expert view definition of organizational purpose as ‘*an organization’s reason for being characterized by significance, aspiration, direction, unification, and motivation.”* Second, we proposed a conceptual model of organizational purpose encompassing its dimensions, antecedents, consequences, mechanisms, and moderators. Third, by explaining how organizational purpose functions and what outcomes may be achieved at intra- and extra-organizational levels and within society, we also contribute to the societal conversation. We conclude with a roadmap for future research and implications for practice.

Our first contribution pertains to the contemporary definition of organizational purpose that presents a clear meaning and interpretation of organizational purpose (Oswald, 2019) through its dimensions: *significance*, *aspiration*, *direction*, *unification*, and *motivation*. Our multifaceted view on the dimensions of organizational purpose differs from previous descriptions and definitions that encompass some of the aspects, but not all, therefore leading to incompleteness (see Table 1). Furthermore, our findings show that a many-sided view on purpose leads to a reconciliation of the previous dichotomous meanings of purpose being either instrumental (e.g., Drucker, 1954) or emotional (Bartlett and Ghoshal, 1994), thereby moving toward a meaning of organizational purpose that can be understood as both instrumental and emotional. In addition, our definition helps to clarify the distinction between organizational purpose (*why* you do business or *what* you are here *for*) and related constructs such as mission (i.e., that *what* you want to achieve in terms of specific activities, specific goals, and a specific timeline), vision (i.e., an imagined future state of what it will be like when the purpose is being lived and the mission accomplished), and (shared) values (i.e., the way *how* you do business and not why). Our study thereby aids in reducing confusion, conflation, and misinterpretations in research and popular literature.

Concerning our second contribution about the proposal of a conceptual model, our study revealed that in line with previous research, *human needs* and *human problems* are antecedents of organizational purpose (Barnard, 1938; Collins and Porras, 2008). By contrast, our interpretation of *founding values* as an antecedent differs from previous research regarding core organizational values (Collins and Porras, 2008). We concluded that *founding values* are intrinsic to purpose (i.e., the values that drive fulfilling certain needs or solving specific problems) and that organizational values are of guidance in behavior (e.g., honesty or integrity). With regard to consequences, our analysis showed to be in line with previous research (e.g., van Ingen et al., 2021), professional reports (e.g., Keller, 2015), and practitioner literature (e.g., Peele et al., 2019). Our contribution lies in the explanation and extension of the intra- and extra-organizational consequences that organizational purpose causes. In considering mechanisms explaining why organizational purpose affects outcomes, our study is in line with previous research on the mechanism of *need fulfillment* in relation to self-determination theory (van Tuin et al., 2020; van Ingen et al., 2021) and on the mechanism of *person–organization fit*, specifically a fit between founding values and individual values (van Ingen et al., 2021). As a contribution to the mechanism of *meaningfulness*, we found that through its significance, organizational purpose at the organizational level complements meaningful work (e.g., Lysova et al., 2019) and the job characteristics model (Hackman and Oldham, 1976) at the job level. Moreover, it can also open up to other research streams, for example in marketing, as meaningfulness seems necessary in marketing for achieving outcomes (Zuo et al., 2019). As a contribution to the mechanism of *need fulfillment*, our study showed that organizational purpose may be considered an organizational resource, thereby complementing conservation of resources theory (Hobfoll, 1989). Furthermore, with *person–organization fit* as a mediator, our study opens research on organizational purpose and outcomes in fields such as employer attractiveness (e.g., Klimkiewicz and Oltra, 2017) and marketing (e.g., Yaniv and Farkas, 2005). Looking at the moderators, our study is in line with previous research on *authenticity* (e.g., Bailey et al., 2017), *communication* (e.g., Nishii and Wright, 2007; Drucker and Maciariello, 2008), and *perception of impact* (Grant, 2007, 2008). Regarding the moderator balance, our study extends research on individual purpose (e.g., Lips-Wiersma, 2002; Lips-Wiersma and Morris, 2009) by addressing organizational purpose to be the collective purpose of individuals who are part of a system that consciously coordinates activities (Barnard, 1938). As such, our findings, on the one hand, differ from previous studies in which organizational purpose focuses solely on the self-interest of the organization and its owners (Friedman, 1970; Jensen, 2002), and on the other hand, differ from studies in which the focus is merely on benefitting others (e.g., Keller, 2015) or satisfying all stakeholders (e.g., Sisodia et al., 2007). As the perception of purpose is subjective, it is likely that the latter is inadequate. Our proposed conceptual model can remove barriers for theory development (Hirsch and Levin, 1999), scale development (MacKenzie et al., 2011), and further empirical research (e.g., van Ingen et al., 2021). Furthermore, we propose organizational purpose to be an umbrella construct (Hirsch and Levin, 1999) that can connect different research streams such as organizational behavior, human resource management, marketing, leadership, and strategy.

Third, our data analysis revealed that every organization has a purpose and that purpose is not a distinctive factor (i.e., organizations can have in essence the same purpose). By contrast, a purpose statement can be a distinctive factor, but not all organizations have a purpose statement (Collins and Porras, 2008). Also, purpose does not solely relate to having a large impact on society for instance in achieving sustainable development goals (Fischer et al., 2019; Mañas-Viniegra et al., 2020). Furthermore, purpose is not to be mistaken with an outcome such as profit (MacKenzie et al., 2011) or a self-interested individual motive (Duska, 1997). Hence, our findings enable people to recognize “purpose-washing” (Oswald, 2019). Our research showed that it is important to keep in mind that whether purpose is perceived as good or bad lies within the eye of the beholder. Furthermore, our analysis showed that the VUCA world we live in, in concurrence with social, economic, and environmental externalities, has not only led to people craving for meaning and purpose in life and work (Ryff et al., 2003) but also led organizations to discover or reevaluate their purpose (Hollensbe et al., 2014).

### Limitations and Future Research

Our findings should be considered in the light of several limitations. Our sample consisted of experts from academia and practice on the topic of organizational purpose. The transferability of our findings is therefore limited. However, our research revealed a global voice from different roles, research, and business streams. Our research and analysis strongly support the importance of organizational purpose in business and society. Future research could incorporate the views of employees, management, customers, suppliers, and other stakeholders. Although our proposed framework shows organizational purpose’s dimensions, antecedents, consequences, moderators, and mechanisms, additional research should more fully develop the understanding of the mechanisms concerning organizational purpose, expanding organizational purpose’s nomological network, including moderators and consequences. Empirical research is needed to test the presented model. As a first step, we suggest developing and validating a measurement instrument to enable empirical research on organizational purpose in relation to consequences, moderators, and mechanisms. Next, after the measurement instrument is established, we suggest using the instrument developed to research how organizational purpose is perceived by organizations’ intra-organizational stakeholders (i.e., corporate or higher management and employees) and extra-organizational stakeholders (i.e., customers, partners, and suppliers) and to investigate whether organizational purpose can prompt psychological mechanisms that directly or indirectly affect stakeholder outcomes at the individual level (e.g., loyalty, trust, and customer engagement) and whether a generational aspect may have influence. These insights can be used to further investigate organizational purpose on multi-stakeholder levels and can help organizations to reflect on their purpose in order to understand its effects on stakeholders. Furthermore, we suggest investigating the effects of organizational purpose at an organizational level in relation to, for instance, performance, organizational culture, organizational identity, and organizational legitimacy. In addition, using our definition of organizational purpose, it might be of interest to evaluate organizations that claim to be purpose-driven on how these organizations, their purposes, and purpose statements are perceived by intra- and extra-organizational stakeholders and to what extent their financial performance differs from non-purpose-driven peer organizations. Furthermore, we suggest doing research revealing which general or highest forms of needs are antecedent to purpose. One might think of the need for safety, belongingness, and reproduction.

### Practical Implications

Our study suggests several practical implications. First and foremost, our study contributes by providing a definition with clear characteristics that may serve as evaluation criteria, enabling management, and practitioners to determine how organizations actually take responsibility in society and whether they act according to their purpose (Duska, 1997; Hollensbe et al., 2014). Second, our findings demonstrated purpose to be both instrumental and emotional, enabling leadership to articulate and convey a purpose statement, set clear goals and objectives, and provide meaningfulness and spirituality in work. This combination may at first sight seem rather revolutionary and breaking with existing paradigms. Third and final, our study helps people to understand that every organization has a purpose and that purpose does not necessarily need to relate to do-gooding or having large impact in societal, environmental, and economical contexts. Doing good is a perception that lies in the eye of the beholder whether that may be an individual or be the norms, values, and cultural aspects in a society. Furthermore, our research showed that even the smallest organizations can have impact on people’s lives.

## Data Availability Statement

The raw data supporting the conclusions of this article will be made available by the authors, without undue reservation.

## Ethics Statement

Ethical approval was not provided for this study on human participants because at the start of the study Nyenrode did not have a formal ethics committee in place, however, we did follow ethical procedures. All 44 participants who took part in this study were informed about the study, its goals, the data collection, and data management procedures. All participants gave written informed consent by signing an informed consent form in which they were informed about who had access to the data, and in which they agreed to the use of their data for this study, the use of pseudonymized quotes in scientific output and the secure storage of their data at Nyenrode Business Universiteit. The patients/participants provided their written informed consent to participate in this study.

## Author Contributions

All authors contributed to the conception and design of the study. RI collected the data. RI, PP, and MR performed the data analysis. RI wrote the first draft of the manuscript. All authors reviewed and edited the manuscript. PP and HR supervised the study. MR co-supervised the study. All authors contributed to the manuscript revision and read and approved the submitted version.

## Conflict of Interest

The authors declare that the research was conducted in the absence of any commercial or financial relationships that could be construed as a potential conflict of interest.
